# The Dental Press Journal of Orthodontics in the social media: a new interactive channel

**DOI:** 10.1590/2177-6709.24.1.014-015.edt

**Published:** 2019

**Authors:** Flavia Artese

**Affiliations:** 1 Universidade do Estado do Rio de Janeiro, Departamento de Odontologia Preventiva e Comunitária (Rio de Janeiro/RJ, Brazil).

The way in which the Internet has changed human communication is unprecedented. Its origins date from the beginning of the 1960’s, with J. C. R. Licklider, at the MIT, and his description of the Galactic Network concept. In parallel, the technology for digital data transfer was being developed independently by different researchers and, in September 1969, the first host computer was connected [to a network] at the University of California (UCLA), creating the ARPANET. Later on, in the 1990’s, at the CERN (*Conseil Européen pour la Recherche Nucléaire*) in Geneva, Tim Berners-Lee developed the *World Wide Web*, together with a browser, that was offered to the public in 1991. And, in this way, the whole world started to be digitally connected.

The Internet is essentially a mechanism for exchanging digital information, and as such, it enables the collaboration and interaction among individuals, through their computers, independently of geographic location. More recently, the interaction concept became more evident in the term coined by Tim O’Reilly in 2004: the Web 2.0. This term describes a second generation of communities that include websites with content created by its users, in what can be considered as an interactive digital community. As tools in this new category of interactions we can include social media websites and their apps.

The Cambridge Dictionary defines social media as an interactive technology mediated by computer, which allows the creation and spreading of information, ideas, professional interests and other expressions through communities or virtual networks. But the influence of social media seems to have a wider range than this. Nowadays, even the choices for presidential elections may be influenced by this means of communications. According to the Washington Post, 35% of voters between 18 and 29 years of age state that social media are the most useful source of information for the presidential campaign. In contrast, voters between 30 and 49 years rank social media in third place, behind cable TV and news websites.^1^

In 1994, Schaffner^2^ described that scientific journals have the following purposes in the academic community: (1) building a knowledge base; (2) communicating information; (3) validating research quality; (4) distributing rewards; and (5) building scientific communities. With these objectives in mind, it is not surprising that the scientific world has been affected by these innovations, either in the speed in which science is being evaluated or in its distribution and promulgation. The long and tiresome searches through the old *Index Medicus*, in the pre-internet era, have been replaced by quick seconds on PubMed. And the clear wish to make information published in scientific journals reach the public much faster has convinced some of the most important journals in the world, such as Nature and Science, to embrace social media.

This new technology allowed the journals to improve their communication, as well as to create scientific communities. The opinion of other researchers can be read almost instantly, opening a new forum for debates and thoughts.^3^ New types of information formats are being created specifically for social media pages, such as visual abstracts, video abstracts, video reviews, and podcasts.^4^ Consequently, a new class of impact measures has been developed, the web-based metrics, which evaluates the amount of mentions (likes, shares, comments, tweets, posts, etc) received by a scientific content in the social media.^5^

 It is believed that this new form of communication may broaden the reach of publications. However, in a recent study, the visibility of orthodontic papers on web platforms was evaluated and no significant effect on the number of citations given to these publications was observed. Despite these results, the authors suggest that orthodontic journals should adjust to this new age to become more active in promoting articles of public interest,^5^ including the general public. And this January the Dental Press Journal of Orthodontics initiated in this new frontier. We created the Instagram and Facebook pages under the title Journal of Orthodontics. We thus invite our readers, editors, reviewers, and all who enjoy our journal to participate actively in our social media.

Looking forward to your likes!

Flavia Artese
